# Stress hyperglycemia is associated with poor prognosis in critically ill patients with cardiogenic shock

**DOI:** 10.3389/fendo.2024.1446714

**Published:** 2024-09-05

**Authors:** Jing Tian, Tao Zhou, Zijuan Liu, Yan Dong, Hongyang Xu

**Affiliations:** Department of Critical Care Medicine, The Affiliated Wuxi People’s Hospital of Nanjing Medical University, Wuxi People’s Hospital, Wuxi Medical Center, Nanjing Medical University, Wux, Jiangsu, China

**Keywords:** stress hyperglycemia ratio (SHR), MIMIC-IV database, cardiogenic shock (CS), mortality, critical care

## Abstract

**Background:**

Stress hyperglycemia is now more common in intensive care unit (ICU) patients and is strongly associated with poor prognosis. Whether this association exists in critically ill patients with cardiogenic shock (CS) is unknown. This study investigated the prognostic relationship of stress hyperglycemia on critically ill patients with CS.

**Methods:**

We included 393 critically ill patients with CS from the MIMIC IV database in this study and categorized the patients into four groups based on quartiles of Stress hyperglycemia ratio (SHR). We assessed the correlation between SHR and mortality using restricted cubic spline analysis and Cox proportional hazards models. The primary outcomes observed were ICU mortality and hospitalization mortality.

**Results:**

The mean age of the entire study population was 68 years, of which 30% were male (118 cases). There was no significant difference between the four groups in terms of age, gender, BMI, and vital signs (P>0.05). There was an increasing trend in the levels of lactate (lac), white blood cell count (WBC), glutamic oxaloacetic transaminase (AST), glucose and Hemoglobin A1C (HbA1c) from group Q1 to group Q2, with the greatest change in patients in group Q4 (P<0.05) and the patients in group Q4 had the highest use of mechanical ventilation, the longest duration of mechanical ventilation, ICU stay and hospital stay. After adjusting for confounders, SHR was found to be strongly associated with patient ICU mortality, showing a U-shaped relationship.

**Conclusion:**

In critically ill patients with CS, stress hyperglycemia assessed by SHR was significantly associated with patient ICU mortality.

## Introduction

Cardiogenic shock (CS) is a highly fatal condition and is a significant cause of morbidity and mortality (1).Based on recent registry data from the United States (US), CS accounts for an estimated 408/100,000 hospitalizations with an average in-hospital mortality rate of 37% (2).There are many factors that predispose CS patients to hyperglycemia, whether they have diabetes or not. Sympathetic stimulation in response to inflammation, poor tissue perfusion due to decreased cardiac output, increased stress response, vasopressin administration, and acquired insulin resistance all contribute to abnormal blood glucose in this setting (3).

Stress-induced hyperglycemia (SIH) is a temporary condition in patients hospitalized for an acute illness that resolves independently after the illness has subsided (4). SIH is common in critically ill patients whether or not they have diabetes on admission and appears to be a marker of disease severity (5). In addition, there is an ongoing controversy about SIH and prognosis (6, 7). Although stress hyperglycemia has been previously demonstrated to be detrimental to the prognosis of cardiovascular disease, however, there is currently no evidence of the prognostic relevance of stress hyperglycemia in patients with CS, especially in critically ill patients (8).

Stress hyperglycemia ratio (SHR), which adjusts for average glycemic status, is recommended for assessing actual blood glucose levels. Several previous studies have proposed that the SHR can be used as an indicator of acute hyperglycemic conditions and as a prognostic indicator of adverse outcomes in critically ill patients (9–11).

Therefore, the aim of this study was to investigate the effect of SIH on the prognosis of critically ill CS patients in the intensive care unit in the hope that clinicians may be alert to stress hyperglycemia in critically ill CS patients and be able to be aware of the possible adverse or concomitant effects of stress hyperglycemia.

## Methods

### Study population

This was a retrospective, observational cohort study, and all relevant data were obtained from the Medical Information Marketplace for Critical Care IV (MIMIC-IV), a publicly accessible database compiled from the electronic health records of Beth Israel Deaconess Medical Center (BIDMC). The author (Jing Tian) obtained the necessary authorization to access the database. It is important to emphasize that our study focused on an analysis of a third-party open-access database that had been approved by the Institutional Review Board (IRB). Therefore, our own institution’s IRB review process was determined to be exempt. In the database, disease diagnoses are based primarily on International Classification of Diseases, Ninth and Tenth Revision (ICD-9 and ICD-10) codes recorded by hospital staff. We identified 1826 as critically ill adult patients diagnosed with CS (codes 78551, R570, T8111XA). Patients missing Hemoglobin A1C (HbA1c) within 24 hours of ICU admission were excluded, thus a total of 393 patients were included. SHR was calculated as SHR = ABG (mg/dL)/(28.7 × HbA1c (%) − 46.7). Four groups were analyzed based on SHR, Q1: ≤0.953, Q2: 0.954-1.231, Q3: 1.232-1.667, Q4: ≥1.668.

### Data collection

Data related to baseline characteristics of patients within 24 hours of ICU admission were extracted from the MIMIC-IV database. These included demographic information such as gender and age as well as basic clinical parameters. And disease severity indicators were retrieved, including Sequential Organ Failure Assessment (Sofa), Simplified Acute Physiology Score II (SAPS II), Systemic Inflammatory Response Syndrome (Sirs), simplified acute physiology score II (Saps II), Oxford acute severity of illness score (Oasis) and Glasgow Coma Scale (Gcs). In addition, vital signs such as heart rate, arterial systolic blood pressure (SBP) arterial diastolic blood pressure (DBP), and general laboratory tests (white blood cell count [WBC], platelets [PLT], albumin [ALB], and creatinine) were recorded. In addition, the patient’s mechanical ventilation, length of ICU stay, and length of hospitalization were calculated. Comorbidities were identified based on recorded ICD-9 codes and included diseases such as hypertension, diabetes mellitus, heart failure (HF), acute myocardial infarction (AMI), chronic obstructive pulmonary disease (COPD), stroke, acute kidney injury (AKI), chronic kidney disease (CKD), and cancer.

For variables with less than 5% missing data, mean interpolation was used as the data completion method. For variables with missing values ranging from 5% to 40%, multivariate interpolation using the chained equations in R (MICE) package provided a robust method of solving for missing data (12).

### Outcomes

The primary observed outcome was the patient’s ICU mortality rate and hospitalization mortality rate, and the secondary outcomes were the patient’s 28-day mortality rate, 90-day mortality rate and 1-year mortality rate.

### Statistical analysis

Patients were categorized into 4 groups based on SHR values. The Kolmogorov-Smirnov test was used to assess whether continuous variables showed normality. Continuous variables that followed a normal distribution were summarized by mean ± standard deviation, while non-normally distributed variables were described using median and interquartile range (IQR). To assess between-group differences for continuous variables, we used Wilcoxon t-tests, and for categorical variables, we used chi-square or Fisher’s exact tests as appropriate. Differences in mortality between the 4 groups were compared using Kaplan-Meier curves and log-rank tests. For evaluating the relationship between SHR and mortality, we conducted univariate and multivariate Cox regression analyses, and the findings were reported in terms of Hazard ratios (HRs) accompanied by their respective 95% CIs. The factors used to construct the Multivariable Cox regression models in this study included: age, gender, MV use, Hypertension, Diabetes, HF, AMI, COPD, Stroke and CKD. Three models were sequentially constructed with or without adjustment for covariates: I) unadjusted; II) age, gender; III) age, sex, BMI, MV use, comorbidities (Hypertension, Diabetes, HF, AMI, COPD, Stroke and CKD). Using Restricted Cubic Spline (RCS) Regression Modeling to Study the Nonlinear Relationship Between SHR and ICU mortality.

A double-sided P < 0.05 was regarded as statistically significant. All statistical analysis was performed by the R software (version 4.0.2) and SPSS 22.0 (IBM SPSS Statistics, Armonk, NY, USA).

## Results

### Baseline characteristics

**Figure 1:**
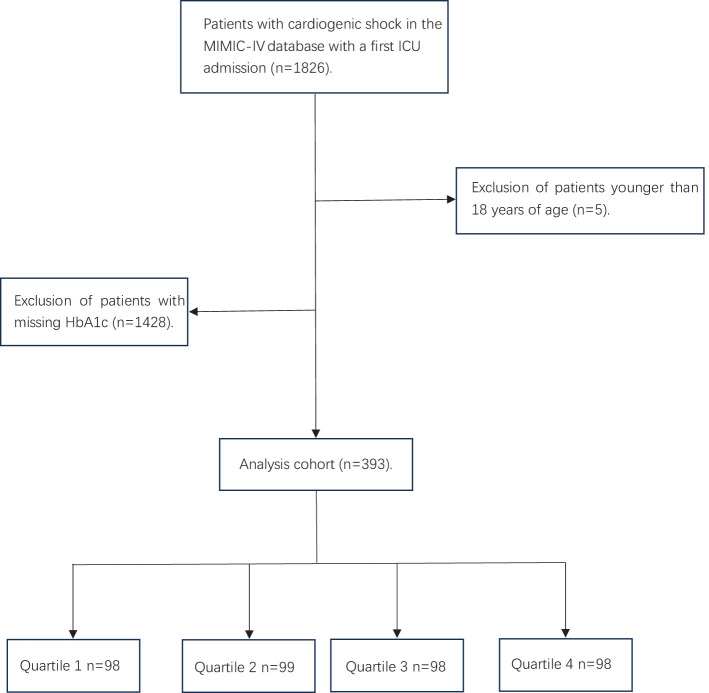
Flow chart of the participants.

A total of 1826 critically ill patients with CS were retrieved from the MIMIC IV database, and 393 patients were entered into the statistical analysis after exclusion of patients younger than 18 years of age and those with missing HbA1c (**Figure 1**). The participants were stratified into four distinct groups (Quartile 1–4) based on their SHR levels: Q1: ≤0.953 N=98, Q2: 0.954-1.231 N=99, Q3: 1.232-1.667 N=98, Q4: ≥1.668 N=98. The mean age of the study cohort was 68 years, of which 118 (30.0%) were male.

Among the four groups, there were no significant differences in age, gender, BMI, and vital signs (P>0.05). Patients in the Q4 group were significantly different from the other three groups in terms of the incidence of diabetes mellitus, AMI, and cancer (P<0.05). Regarding the severity of disease scores, there were significant differences in all scores except for the GCS score, which was not statistically different. Patients’ lactate, WBC, glutamic oxaloacetic transaminase (AST), glucose and HbA1c levels showed an increasing trend from group Q1 to Q4, and the greatest change was observed in patients in group Q4 (P<0.05). In addition, the patients in group Q4 had the highest utilization of mechanical ventilation and the longest duration of mechanical ventilation, ICU stay, and hospitalization compared to the other three groups (P<0.05). Specific baseline data can be found in **Table 1**.

**Table 1:** Based on the baseline characteristics of the SHR quartiles.

	Q1, N=98	Q2, N = 99	Q3, N = 98	Q4, N = 98	P-value
Age, years	68 (56- 77)	68 (57-75)	67 (59-77)	68 (60-77)	0.879
Sex, %					0.215
Male	28 (28.57%)	24 (24.24%)	29 (29.59%)	37 (37.76%)	
Female	70 (71.43%)	75 (75.76%)	69 (70.41%)	61 (62.24%)	
BMI	29.3 (26.0-32.0)	28.1 (25.7-31.2)	28.0 (25.9-30.3)	28.9 (25.3-31.7)	0.269
Comorbidities, %					
Hypertension	25 (25.51%)	26 (26.26%)	31 (31.63%)	32 (32.65%)	0.592
Diabetes	47 (47.96%)	31 (31.31%)	26 (26.53%)	43 (43.88%)	0.005
HF	84 (85.71%)	79 (79.80%)	75 (76.53%)	72 (73.47%)	0.184
AMI	32 (32.65%)	37 (37.37%)	47 (47.96%)	55 (56.12%)	0.004
COPD	12 (12.24%)	8 (8.08%)	5 (5.10%)	13 (13.27%)	0.185
Stroke	12 (12.24%)	8 (8.08%)	11 (11.22%)	7 (7.14%)	0.569
AKI	63 (64.29%)	62 (62.63%)	60 (61.22%)	64 (65.31%)	0.938
CKD	28 (28.57%)	31 (31.31%)	24 (24.49%)	20 (20.41%)	0.325
Cancer	4 (4.08%)	15 (15.15%)	10 (10.20%)	16 (16.33%)	0.029
Severity of illness					
Sofa	6.0 (3.0-8.8)	6.0 (3.0-8.0)	7.0 (4.0-9.0)	8.0 (6.0- 11.0)	<0.001
Aps iii	46 (37-59)	48 (38-59)	50 (38-62)	58 (46-72)	<0.001
Sirs	2.0(2.0-3.0)	3.0 (2.0-3.0)	3.0 (3.0-3.0)	3.00 (3.0-3.8)	<0.001
Saps ii	36 (27-47)	38 (29-46)	41 (32-50)	46 (37-55)	<0.001
Oasis	31 ± 9	33 ± 9	34 ± 8	37 ± 9	<0.001
Gcs	15.0 (14.0-15.0)	15.0 (14.0-15.0)	15.0 (14.1-15.0)	15.0 (14.0-15.0)	0.500
Vital signs					
Heart rate, bpm	90 (80-101)	91 (76-106)	89 (78-104)	92 (78-109)	0.743
SBP, mmHg,	111 (102-120)	109 (100-123)	109 (101-121)	110 (98-119)	0.905
DBP, mmHg,	59 (54-67)	61 (50-69)	61 (54-72)	59 (51-66)	0.583
Mean arterial pressure, mmHg,	79 (67-88)	79 (71, 87)	80 (68, 90)	77 (67-87)	0.560
Laboratory tests					
Lac, mmol/L	1.90 (1.30-2.90)	1.80 (1.30-2.35)	2.00 (1.50-3.25)	2.80 (1.70-4.28)	<0.001
WBC, K/uL	9.6 (7.3-13.0)	11.6 (8.7-14.7)	13.8 (10.7-16.7)	16.8 (12.6-21.2)	<0.001
HB, g/dL	11.61 ± 2.50	11.61 ± 2.45	11.76 ± 2.71	11.98 ± 2.60	0.724
ALB, g/dL	3.26 (2.90, 3.50)	3.40 (2.90-3.60)	3.20 (2.90-3.53)	3.28 (2.83-3.50)	0.8432
ALT, IU/L	67 (25-186)	47 (23-106)	52 (28-127)	88 (30-250)	0.118
AST, IU/L	95 (28-279)	68 (27-261)	116 (45-212)	152 (43-438)	0.047
Glucose, mg/dL	106 (93-133)	128 (118-154)	172 (154-193)	281 (223-351)	<0.001
HbA1c, %	6.30 (5.80-7.980	5.70 (5.40-6.70)	5.80 (5.40-6.48)	6.00 (5.53-6.30)	<0.001
MV, %	86 (87.76%)	92 (92.93%)	90 (91.84%)	89 (90.82%)	0.624
MV time, hours	46 (15, 122)	87 (42, 163)	61 (30-150)	76 (38-153)	0.012
ICU LOS, days	4 (2-9)	6 (4-12)	5 (3-10)	5 (3-9)	0.026
Hospital LOS, days	13 (7-21)	16 (8-25)	15 (7-24)	9 (5-17)	0.002

HF, Heart failure; AMI, Acute myocardial infarction; COPD, Chronic obstructive pulmonary disease; AKI, Acute kidney injury; CKD, Chronic kidney disease; Sofa, Sequential Organ Failure Assessment; SAPS II, Simplified Acute Physiology Score II; Sirs, Systemic Inflammatory Response Syndrome; Saps II, Simplified acute physiology score II; Oasis, Oxford acute severity of illness score; Gcs, Glasgow Coma Scale; SBP, Arterial systolic blood pressure; DBP, arterial diastolic blood pressure; Lac, Lactate; WBC, White blood cell count; PLT, Platelets; Hb; Hb Hemoglobin; ALB, Albumin; ALT, Alanine aminotransferase; AST, Glutamine aminotransferase; HbA1c, Hemoglobin A1C; MV, mechanical ventilation.

### Association between SHR quartiles and mortality rates

**Figure 2:**
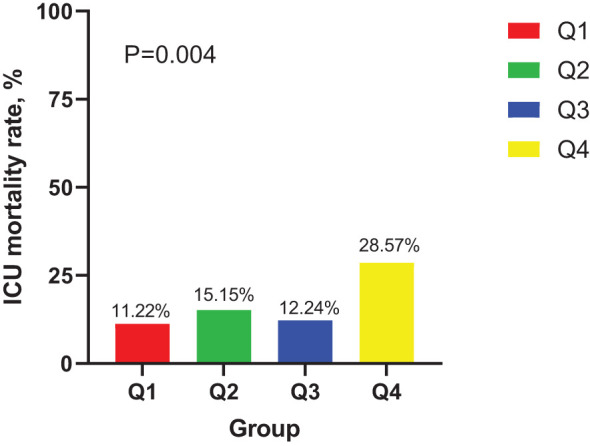
Distribution of ICU mortality in four groups of patients.

**Figure 3:**
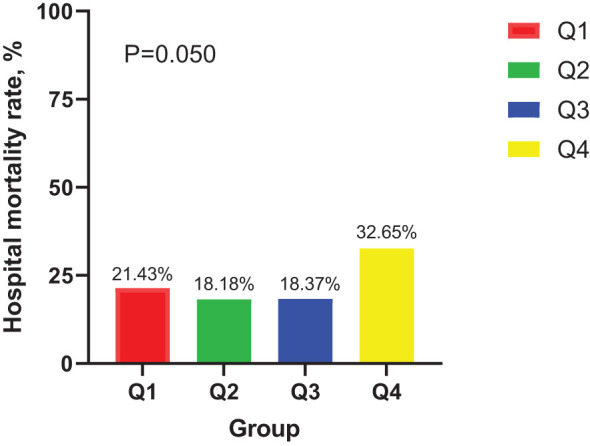
Distribution of hospitalized mortality in four groups of patients.

**Figure 4:**
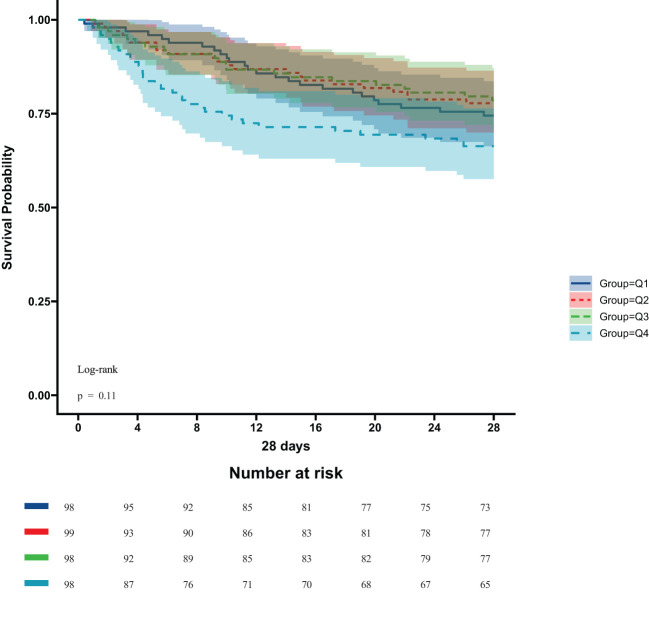
Kaplan−Meier survival curves for 28-day mortality in four groups of patients.

**Figure 5:**
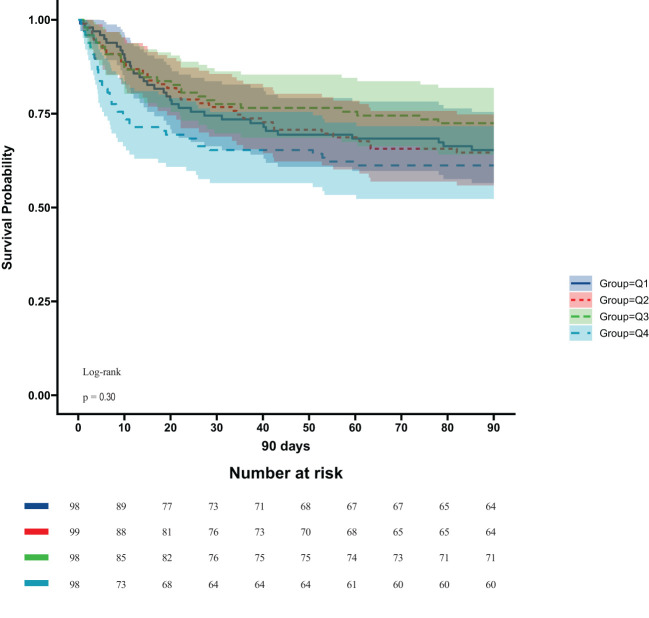
Kaplan−Meier survival curves for 90-day mortality in four groups of patients.

**Figure 6:**
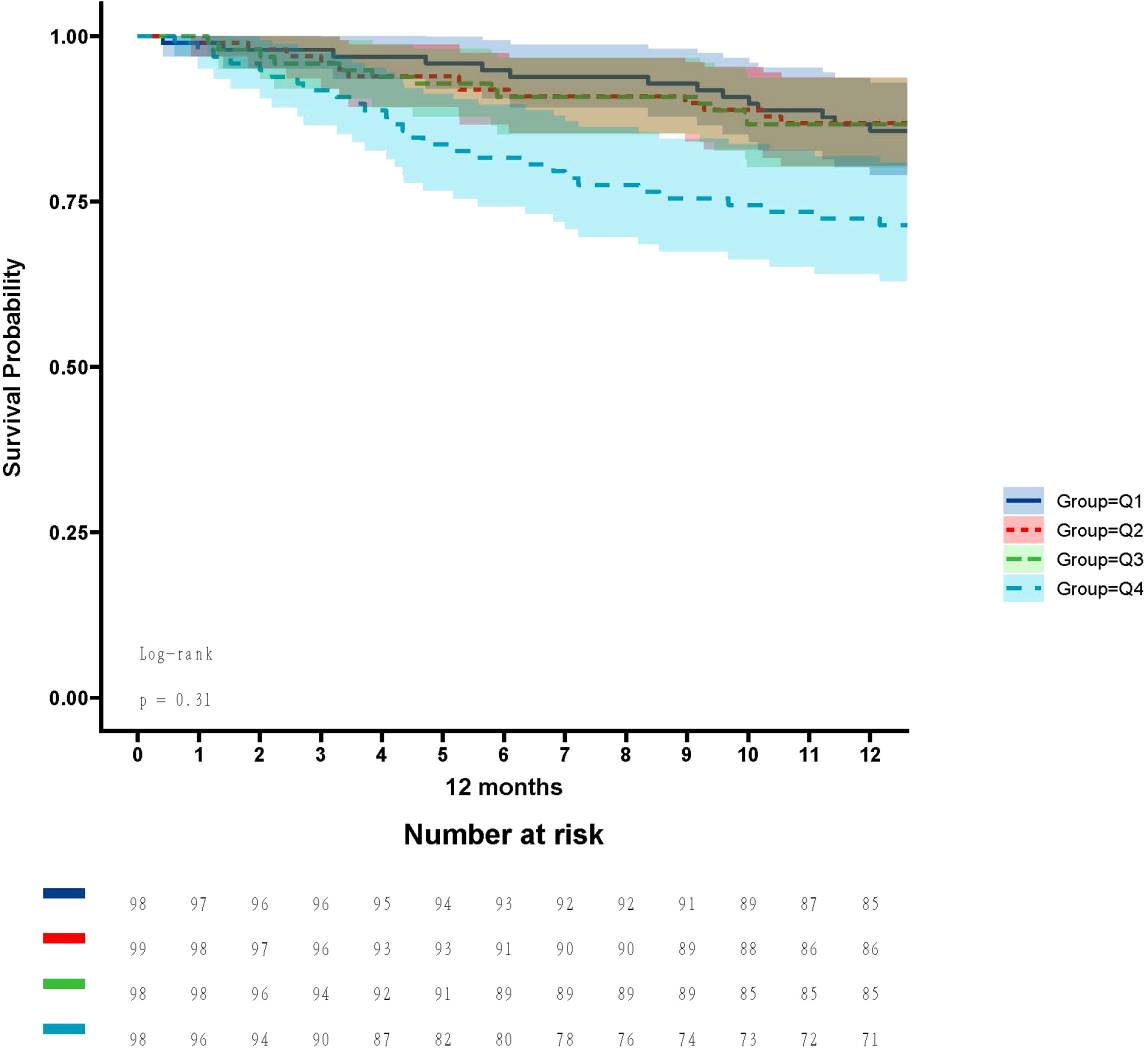
Kaplan−Meier survival curves for 1-year mortality in four groups of patients.

In the ICU stays, the mortality rate for patients in group Q4 notably exceeded that of groups Q1, Q2, and Q3 (28.57% vs.11.22%, 15.15%, 12.24%, P < 0.05) (**Figure 2**). Regarding mortality rates during hospital stays, even though the outcomes were less favorable for patients in group Q4, the variance was not markedly significant (P=0.05) (**Figure 3**). Based on the Kaplan−Meier survival curves, participants in the Q4 group showed the utmost mortality rates on days 28, 90, and one year, yet these were not statistically significant when compared to the other three groups (P>0.05) (**Figures 4**–**6**).

### Factors associated with ICU mortality

**Table 2** summarizes the univariate and multivariate analyses of ICU mortality in critically ill patients with CS. Age, HF, Aps iii score, duration of mechanical ventilation, and SHR were significantly associated with the occurrence of ICU death in patients with CS (P<0.05).

**Table 2:** Identification of risk factors for ICU mortality in critically ill patients with CS: a Cox multivariate analysis.

Characteristic	Univariable			Multivariable		
	HR	95% CI	P-value	HR	95% CI	P-value
age	1.040	1.020, 1.060	<0.001	1.043	1.005, 1.083	0.027
sex						
Male	-	-	-	-	-	-
Female	0.692	0.416, 1.153	0.158	-	-	-
BMI	0.971	0.931, 1.014	0.182	-	-	-
Hypertension	1.015	0.594, 1.736	0.956	-	-	-
Diabetes	0.611	0.373, 0.999	0.050	-	-	-
HF	1.766	1.057, 2.953	0.030	4.007	1.835, 8.751	<0.001
AMI	0.663	0.404, 1.088	0.104	-	-	-
COPD	1.308	0.562, 3.048	0.533	-	-	-
Stroke	2.802	0.683, 11.500	0.153	-	-	-
AKI	0.981	0.560, 1.721	0.948	-	-	-
CKD	0.661	0.391, 1.118	0.123	-	-	-
Cancer	0.595	0.308, 1.151	0.123	-	-	-
Sofa	1.113	1.045, 1.186	<0.001	0.990	0.840, 1.167	0.906
Aps iii	1.021	1.011, 1.031	<0.001	1.045	1.019, 1.072	<0.001
Sirs	1.068	0.797, 1.431	0.660	-	-	-
Saps ii	1.045	1.028, 1.062	<0.001	1.001	0.955, 1.050	0.954
Oasis	1.061	1.032, 1.091	<0.001	1.013	0.957, 1.074	0.649
Gcs	0.959	0.885, 1.039	0.304	-	-	-
Heart rate	0.992	0.981, 1.004	0.183	-	-	-
Systolic blood pressure	0.991	0.979, 1.002	0.121	-	-	-
Diastolic blood pressure	0.979	0.963, 0.995	0.011	0.996	0.970, 1.023	0.782
Mean arterial pressure	0.994	0.987, 1.001	0.094	-	-	-
Lac	1.080	1.008, 1.158	0.029	0.993	0.866, 1.138	0.915
WBC	1.021	0.989, 1.054	0.206	-	-	-
HB	1.013	0.920, 1.117	0.787	-	-	-
ALB	1.021	0.989, 1.054	0.206	-	-	-
ALT	1.000	1.000, 1.000	0.585			
AST	1.000	1.000, 1.000	0.582	-	-	-
Glucose	1.508	0.919, 2.473	0.104	1.971	1.060, 3.664	0.032
HbA1c	0.981	0.848, 1.134	0.793	-	-	-
MV use	5.621	2.842, 11.118	<0.001	2.193	0.860, 5.588	0.100
MV time	0.993	0.991, 0.996	<0.001	0.992	0.988, 0.995	<0.001
SHR	1.513	1.150, 1.991	0.003	10.415	1.567, 69.219	0.015

HR, Hazard ratio; CI, Confidence interval.

HF, Heart failure; AMI, Acute myocardial infarction; COPD, Chronic obstructive pulmonary disease; AKI, Acute kidney injury; CKD, Chronic kidney disease; Sofa, Sequential Organ Failure Assessment; SAPS II, Simplified Acute Physiology Score II; Sirs, Systemic Inflammatory Response Syndrome; Saps II, Simplified acute physiology score II; Oasis, Oxford acute severity of illness score; Gcs, Glasgow Coma Scale; SBP, Arterial systolic blood pressure; DBP, arterial diastolic blood pressure; Lac, Lactate; WBC, White blood cell count; PLT, Platelets; Hb; Hb Hemoglobin; ALB, Albumin; ALT, Alanine aminotransferase; AST, Glutamine aminotransferase; HbA1c, Hemoglobin A1C; MV, mechanical ventilation.

### Association between the SHR and ICU mortality

**Figure 7:**
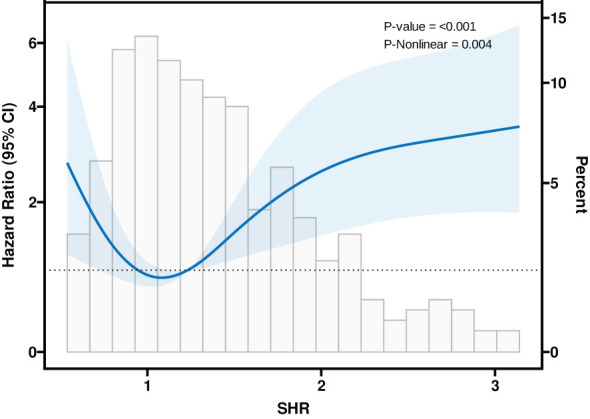
Restricted cubic spline curve for the SHR Hazard ratio.

**Table 3** summarizes the three models constructed to explore the relationship between SHR and mortality in CS critically ill patients in the ICU, namely, Model I, Model II, and Model III. In Model I, no adjustment was made for confounders; in Model II, patients were adjusted for sex, age, and body mass index; and in Model III, adjustments were made for age, sex, BMI, age, gender, BMI, MV use, Hypertension, Diabetes, HF, AMI, COPD, Stroke and CKD. The risk of death during ICU hospitalization in CS critically ill patients was significantly associated with SHR in all three different models (P < 0.05). In **Figure 7**, a nonlinear association between SHR and patients’ ICU mortality was demonstrated (P < 0.05), and the association between SHR and ICU mortality in critically ill CS patients was U-shaped.

**Table 3:** The association between SHR and ICU mortality by Cox proportional-hazard regression.

	HR	95%CI	P-value
SHR (Model I)	1.513	1.150, 1.991	0.003
SHR (Model II)	1.554	1.165, 2.075	0.003
SHR (Model III)	1.511	1.124, 2.030	0.006

HR, Hazard ratio; CI, Confidence interval.

Mode II adjusted for none;

Mode III adjusted for age, gender and BMI;

Model III adjusted for age, gender, BMI, MV use, Hypertension, Diabetes, HF, AMI, COPD, Stroke, CKD.

## Discussion

We retrospectively analyzed 393 critically ill patients with CS from the MIMIC IV database to examine the effect of SHR on intensive care unit and in-hospital mortality. Our study found that SHR was significantly correlated with intensive care unit mortality in a U-shaped pattern, with a nonlinear association between the two groups. According to the Multivariable Cox regression models, there was a significant effect of SHR on ICU mortality in either Model I, Model II, or Model III. In addition, SHR had no significant effect on the prognosis of CS patients after 28 days, 90 days, or 1 year. Therefore, SHR can effectively predict the prognosis of patients with CS during ICU transition.

Stress hyperglycemia is usually a transient hyperglycemia that occurs during illness and may be related to the patient’s basal glucose tolerance, the type and severity of the disease, and the stage of the disease (13). The SHR is more predictive of a patient’s prognosis than hyperglycemia on admission (14). In a large retrospective study, ROBERTS et al. found that a better biomarker of critical illness was the SHR, as this indicator not only controlled for background hyperglycemia in individual patients, but was an independent predictor of critical illness in all patients with good glycemic status (15). In a retrospective study, Chong Zhang et al. found that higher SHR was associated with ICU death and long-term all-cause mortality in 3,887 critically ill patients and that SHR, as a novel and validated biomarker, had an incremental effect on a variety of disease scores in predicting ICU all-cause mortality (16). The development of stress hyperglycemia is not uncommon in the ICU. Previous studies have shown that the incidence of stress hyperglycemia increases significantly within 48 hours of ICU admission in at least 50% of patients (17). It may be related to the following explanations: 1. Glucose, as a proinflammatory mediator, increases and enhances transcription factors that regulate genes encoding proinflammatory mediators making further promotion of inflammatory cytokine production and contributing to the process of inflammation development (18). 2. Stress hyperglycemia reduces endothelial nitric oxide, leading to insufficient perfusion of organs secondary to vasoconstriction, which further impairs the microcirculation (18, 19). 3. Stress hyperglycemia increases the infection risk, and increased glucose influx into immune cells leads to glucose toxicity. Impairs neutrophil function, promotes lymphocyte apoptosis and inhibits T-cell proliferation (20).

There is a lack of clarity regarding the relationship between SHR and outcomes in critically ill patients with CS. Previous studies have shown a significant effect of SHR on cardiovascular events. Stress hyperglycemia is associated with inflammation, oxidative stress, microvascular injury, endothelial dysfunction, and a prothrombotic state, which can lead to impaired myocardial blood flow and decreased cardiac function (21). Gao S et al. included 1179 patients with nonobstructive coronary myocardial infarction to investigate the relationship between SHR and adverse cardiovascular events (all-cause mortality, nonfatal myocardial infarction, stroke, revascularization, and hospitalization for unstable angina or heart failure), and demonstrated that SHR independently determines the risk of cardiovascular events after nonobstructive coronary myocardial infarction (22). In addition, another study showed that an elevated SHR was independently associated with an increased risk of all-cause composite events, cardiovascular mortality, and readmission due to HF compared with patients with heart failure who had a lower SHR (23). CS is a life-threatening syndrome of cardiac insufficiency and systemic under perfusion with increasing morbidity and persistently high mortality (24, 25). Being related to both microcirculation and cardiac function, there is a need to explore SHR and the prognosis of critically ill patients with CS.

It is always known that cardiogenic shock is a low cardiac output state that leads to life-threatening end-organ underperfusion and hypoxia, seriously threatening the patient’s prognosis (26). Indeed, it is now well established that cardiogenic shock can lead to acute and subacute disturbances of the entire circulatory system, including the peripheral vascular system. Death and inadequate perfusion of vital organs remain clinical features (25). On the one hand, peripheral vasoconstriction may improve coronary and peripheral perfusion at the expense of increased afterload, allowing cardiac compensation for eventual cardiac failure; on the other hand, systemic inflammation triggered by acute cardiac injury may induce pathologic vasodilation (27). Endothelial damage and high levels of NO are involved, and oxidative stress occurs when the production of internal ROS (reactive oxygen species) exceeds their degradation through antioxidant defenses, and elevated ROS lead to cellular damage through oxidation, disrupting intravascular homeostasis by interfering with NO, which on the one hand affects myocardial hypertrophy to the point of fibrosis, ventricular contractile dysfunction, and even pump failure, and on the other hand leads to vasoconstriction, platelet aggregation, and ineffective oxygen delivery (28, 29). Many factors in the ICU predispose CS patients to hyperglycemia, regardless of the presence or absence of preexisting diabetes. Sympathetic stimulation in the presence of an inflammatory response, reduced cardiac output leading to poor tissue perfusion, excessive stress response, vasopressor utilization, and acquired insulin resistance all contribute to aggravated cardiac dysfunction in this setting (3). Whereas the dangers of hyperglycemia have been elucidated above, and as suggested by multifactorial analysis and restrictive cubic bar graphs, SHR was found to be indeed an independent risk factor for the prognosis of patients with CS during the ICU, and exceeding a certain range can seriously threaten the survival of the patient.

Our study focuses on emphasizing the relationship between critically ill patients with cardiogenic shock and SHR, and the attention is on the association between the changes in the patients’ conditions that exist during the transition period in the ICU and SHR to provide some guidance for the subsequent treatment. In addition, in the statistical analysis, when the patients safely passed through the ICU period, there was no difference in their late prognosis. This is a point of difference with the study of Li Le’s team (10). Although their data also used the public resource MIMIC IV, they emphasized critically ill patients with all diseases and did not elaborate on the cardiac circulation, and their focus was on all-cause mortality rather than ICU mortality, with emphasis on the relationship between overall mortality and SHR in critically ill patients.

Our findings emphasize the importance of estimating stress hyperglycemia for predicting prognosis in patients with CS. SHR was significantly correlated with ICU mortality in critically ill patients with CS. When adjusted for confounders, SHR still maintained its association with ICU mortality. Although the pathophysiologic mechanism of stress hyperglycemia and CS is unclear, this study reminds clinicians of the importance of glycemic control in patients with CS.

### Limitations

There are several limitations of our study that are worth considering. First, the data were obtained from the MIMIC IV database, and selection bias could not be avoided. Second, we could only observe the phenomena and could not study the pathophysiological mechanisms in detail, and finally, the lack of information on other inflammatory markers, the use of glucose-lowering medications, and serial changes in blood glucose and glycosylated hemoglobin during the hospitalization and the subsequent follow-up may affect the long-term outcome, thus limiting our overall understanding of the poor prognosis. Additional immunological experiments and multicenter studies on this easily available indicator are needed and are important for early prediction of deterioration in patients with severe CS and will certainly benefit clinical care.

## Conclusion

In conclusion, this study revealed a U-shaped association between SHR and ICU mortality in patients with CS. The results emphasize that SHR has a significant predictive value for ICU mortality and more future studies to explore the relationship between SIH and CS.

## Data Availability

The original contributions presented in the study are included in the article/supplementary material. Further inquiries can be directed to the corresponding author.

## References

[1] Reyentovich A Barghash MH Hochman JS. Management of refractory cardiogenic shock. Nat Rev Cardiol. (2016) 13:481–92. doi: 10.1038/nrcardio.2016.96 27356877

[2] Osman M Syed M Patibandla S Sulaiman S Kheiri B Shah MK. Fifteen-year trends in incidence of cardiogenic shock hospitalization and in-hospital mortality in the United States. J Am Heart Assoc. (2021) 10:e021061. doi: 10.1161/JAHA.121.021061 34315234 PMC8475696

[3] Nair RM Chawla S Mentias A Saleem T Vural A Ko T. Glycemic patterns and impact of early hyperglycaemia in patients with cardiogenic shock on mechanical circulatory support. Eur Heart J Acute Cardiovasc Care. (2023) 12:328–35. doi: 10.1093/ehjacc/zuad032 37010099

[4] Russo MP Fosser SNM Elizondo CM Giunta DH Fuentes NA Grande-Ratti MF. In-hospital mortality and glycemic control in patients with hospital hyperglycemia. Rev Diabetic Stud. (2021) 17:50–6. doi: 10.1900/RDS.2021.17.50 PMC9380085 34852895

[5] Corstjens AM van der Horst ICC Zijlstra JG Groeneveld AB Zijlstra F Tulleken JE. Hyperglycaemia in critically ill patients: marker or mediator of mortality? Crit Care (London England). (2006) 10:216. 10.1186/cc4957 PMC1550943 16834760

[6] Marik PE Bellomo R. Stress hyperglycemia: an essential survival response. Crit Care. (2013) 17:305. doi: 10.1186/cc12514 23470218 PMC3672537

[7] Badawi O Waite MD Fuhrman SA Zuckerman IH. Association between intensive care unit–acquired dysglycemia and in-hospital mortality*. Crit Care Med. (2012) 40:3180–8. doi: 10.1097/CCM.0b013e3182656ae5 22971590

[8] Huang YW An YH Yin XS Li ZP. Association of the stress hyperglycemia ratio and clinical outcomes in patients with cardiovascular diseases: a systematic review and meta-analysis. Eur Rev Med Pharmacol Sci. (2022) 26:9258–69. 10.26355/eurrev_202212_30679 36591838

[9] Cui K Fu R Yang J Xu H Yin D Song W. The impact of fasting stress hyperglycemia ratio, fasting plasma glucose and hemoglobin A1c on in-hospital mortality in patients with and without diabetes: findings from the China acute myocardial infarction registry. Cardiovasc Diabetol. (2023) 22:165. doi: 10.1186/s12933-023-01868-7 37403082 PMC10320917

[10] Li L Zhao M Zhang Z Zhou L Zhang Z Xiong Y. Prognostic significance of the stress hyperglycemia ratio in critically ill patients. Cardiovasc Diabetol. (2023) 22:275. doi: 10.1186/s12933-023-02005-0 37833697 PMC10576399

[11] Yan F Zhao L Quan X Zhu J. Association between stress hyperglycemia ratio and diabetes mellitus mortality in American adults: a retrospective cohort study and predictive model establishment based on machine learning algorithms (NHANES 2009-2018). Diabetol Metab Syndr. (2024) 16:79. doi: 10.1186/s13098-024-01324-w 38566220 PMC10986058

[12] Zhang Z. Multiple imputation with multivariate imputation by chained equation (MICE) package. Ann Trans Med. (2016) 4:30. 10.3978/j.issn.2305-5839.2015.12.63 PMC4731595 26889483

[13] Dungan KM Braithwaite SS Preiser J. Stress hyperglycaemia. Lancet. (2009) 373:1798–807. doi: 10.1016/S0140-6736(09)60553-5 PMC3144755 19465235

[14] Rau CS Wu SC Chen YC Chien PC Hsieh HY Kuo PJ. Stress-induced hyperglycemia in diabetes: A cross-sectional analysis to explore the definition based on the trauma registry data. Int J Environ Res Public Health. (2017) 14:1527. doi: 10.3390/ijerph14121527 29215581 PMC5750945

[15] Roberts GW Quinn SJ Valentine N Alhawassi T O'Dea H Stranks SN. Relative hyperglycemia, a marker of critical illness: introducing the stress hyperglycemia ratio. J Clin Endocrinol Metab. (2015) 100:4490–7. doi: 10.1210/jc.2015-2660 26485219

[16] Zhang C Shen HC Liang WR Ning M Wang ZX Chen Y. Relationship between stress hyperglycemia ratio and allcause mortality in critically ill patients: Results from the MIMIC-IV database. Front Endocrinol (Lausanne). (2023) 14:1111026. doi: 10.3389/fendo.2023.1111026 37077351 PMC10106677

[17] Plummer MP Bellomo R Cousins CE Annink CE Sundararajan K Reddi BA. Dysglycaemia in the critically ill and the interaction of chronic and acute glycaemia with mortality. Intensive Care Med. (2014) 40:973–80. doi: 10.1007/s00134-014-3287-7 24760120

[18] Kajbaf F Mojtahedzadeh M Abdollahi M. Mechanisms underlying stress-induced hyperglycemia in critically ill patients. Therapy. (2007) 4:97–106. doi: 10.2217/14750708.4.1.97

[19] Yuan T Yang T Chen H Fu D Hu Y Wang J. New insights into oxidative stress and inflammation during diabetes mellitus-accelerated atherosclerosis. Redox Biol. (2019) 20:247–60. doi: 10.1016/j.redox.2018.09.025 PMC6205410 30384259

[20] Matias CN Lima V Teixeira HM Souto FR Magalhães V. Hyperglycemia increases the complicated infection and mortality rates during induction therapy in adult acute leukemia patients. Rev Bras Hematol Hemoter. (2013) 35:39–43. doi: 10.5581/1516-8484.20130013 23580883 PMC3621634

[21] Timmer JR Ottervanger JP de Boer MJ Dambrink JH Hoorntje JC Gosselink AT. Hyperglycemia is an important predictor of impaired coronary flow before reperfusion therapy in ST-segment elevation myocardial infarction. J Am Coll Cardiol. (2005) 45:999–1002. doi: 10.1016/j.jacc.2004.12.050 15808754

[22] Gao S Huang S Lin X Xu L Yu M. Prognostic implications of stress hyperglycemia ratio in patients with myocardial infarction with nonobstructive coronary arteries. Ann Med. (2023) 55:990–9. doi: 10.1080/07853890.2023.2186479 PMC10795641 36896774

[23] Huang H Liu Q Zhu L Zhang Y Lu X Wu Y. Prognostic value of preoperative systemic immune-inflammation index in patients with cervical cancer. Sci Rep. (2019) 23:67. doi: 10.1038/s41598-019-39150-0 PMC6397230 30824727

[24] Chioncel O Parissis J Mebazaa A Thiele H Desch S Bauersachs J. Epidemiology, pathophysiology and contemporary management of cardiogenic shock – a position statement from the Heart Failure Association of the European Society of Cardiology. Eur J Heart Failure. (2020) 22:1315–41. doi: 10.1002/ejhf.1922 32469155

[25] van Diepen S Katz JN Albert NM Henry TD Jacobs AK Kapur NK. Contemporary management of cardiogenic shock: A scientific statement from the american heart association. Circulation. (2017) 136:e232–68. doi: 10.1161/CIR.0000000000000525 28923988

[26] Thiele H Zeymer U Neumann FJ. Intraaortic balloon support for myocardial infarction with cardiogenic shock. N Engl J Med. (2012) 367:1287–96. doi: 10.1056/NEJMoa1208410 22920912

[27] Hochman JS. Cardiogenic shock complicating acute myocardial infarction: expanding the paradigm. Circulation. (2003) 107:2998–3002. doi: 10.1161/01.CIR.0000075927.67673.F2 12821585

[28] Wold LE Ceylan-Isik AF Fang CX Yang X Li SY Sreejayan N. Metallothionein alleviates cardiac dysfunction in streptozotocin-induced diabetes: Role of Ca2+ cycling proteins, NADPH oxidase, poly (ADP-Ribose) polymerase and myosin heavy chain isozyme. Free Radical Biol Med. (2006) 40:1419–29. doi: 10.1016/j.freeradbiomed.2005.12.009 16631532

[29] Rao SV Jollis JG Harrington RA Granger CB Newby LK Armstrong P. Relationship of blood transfusion and clinical outcomes in patients with acute coronary syndromes. JAMA. (2004) 292:1555–62. doi: 10.1001/jama.292.13.1555 15467057

